# Emerging molecular and environmental biomarkers of shrimp allergy in African Americans in the US

**DOI:** 10.3389/falgy.2026.1817101

**Published:** 2026-04-10

**Authors:** Tanmoy Mondal, Dalyngs Duvelsaint, Kingston Griffin, McKenzie Williams, Oluwaseyitodun Johnson, Zara Campbell, Carla M. Davis

**Affiliations:** Department of Pediatrics and Child Health, Howard University College of Medicine, Washington, DC, United States

**Keywords:** African Americans, biomarkers, multi omics, shrimp allergy, tropomyosin

## Abstract

Shrimp allergy (SA), a major cause of food-induced anaphylaxis, represents a disproportionate and under-characterized burden among African American (AA) populations in the United States. Unlike many childhood food allergies, SA is often persistent and commonly presents in adolescence or adulthood, suggesting a role for cumulative environmental exposures in disrupting oral tolerance. A key diagnostic challenge in AA communities is the high prevalence of IgE sensitization to shrimp tropomyosin (Pen a 1), which shares strong structural homology with cockroach and house dust mite tropomyosins, leading to frequent cross-reactive but clinically irrelevant sensitization in urban settings. This review critically examines molecular and environmental biomarkers of SA with a focus on AA populations. We assess the limitations of extract-based IgE testing and component-resolved diagnostics, highlighting how single-component assays may overestimate true clinical allergy. We emphasize the added value of functional assays, particularly the basophil activation test, in distinguishing sensitization from challenge-confirmed allergies. Mechanistically, we discuss how chronic exposure to indoor arthropod allergens, air pollution, and socioenvironmental stressors may drive epithelial barrier dysfunction, IL-33 release, and amplification of type 2 immune pathways, lowering reaction thresholds and influencing disease persistence. We identify key gaps, including limited oral food challenge–confirmed data and underrepresentation of AA cohorts. Finally, we propose equity-centered, integrative research frameworks combining molecular diagnostics, functional assays, environmental assessment, and multi-omics to improve diagnostic precision and advance clinical equity in SA care.

## 1 Introduction

Shrimp allergy (SA) is among the most common food allergies (FAs) in adolescents and adults and represents a leading cause of food-induced anaphylaxis worldwide (1–3). While epidemiologic surveys often group crustacean and mollusk allergies under the umbrella of “shellfish allergy,” shrimp represents the predominant driver of shellfish-related sensitization, clinical reactions, and anaphylaxis in adolescents and adults. In contrast to many childhood FAs such as milk or egg, which frequently resolve with age, SA is typically persistent and often presents during adolescence or adulthood after years or decades of prior tolerance (3–5). This late-onset phenotype is clinically consequential, as shellfish allergy/SA is associated with a high burden of severe reactions, emergency department utilization, and sustained dietary restriction, contributing to impaired quality of life in affected individuals (6).

**Figure 1:**
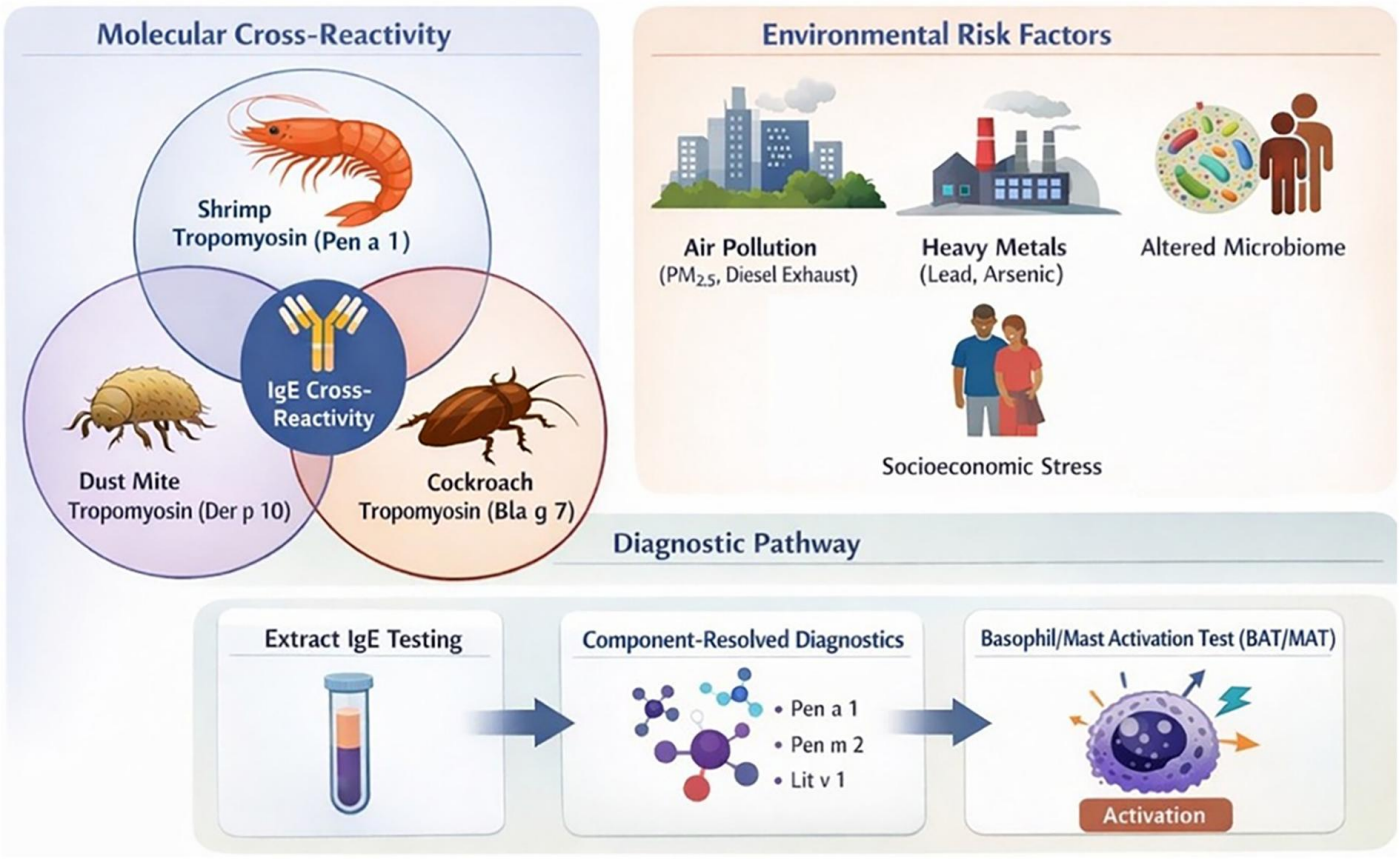
Shrimp allergy in African Americans: molecular and environmental factors.

**Figure 2:**
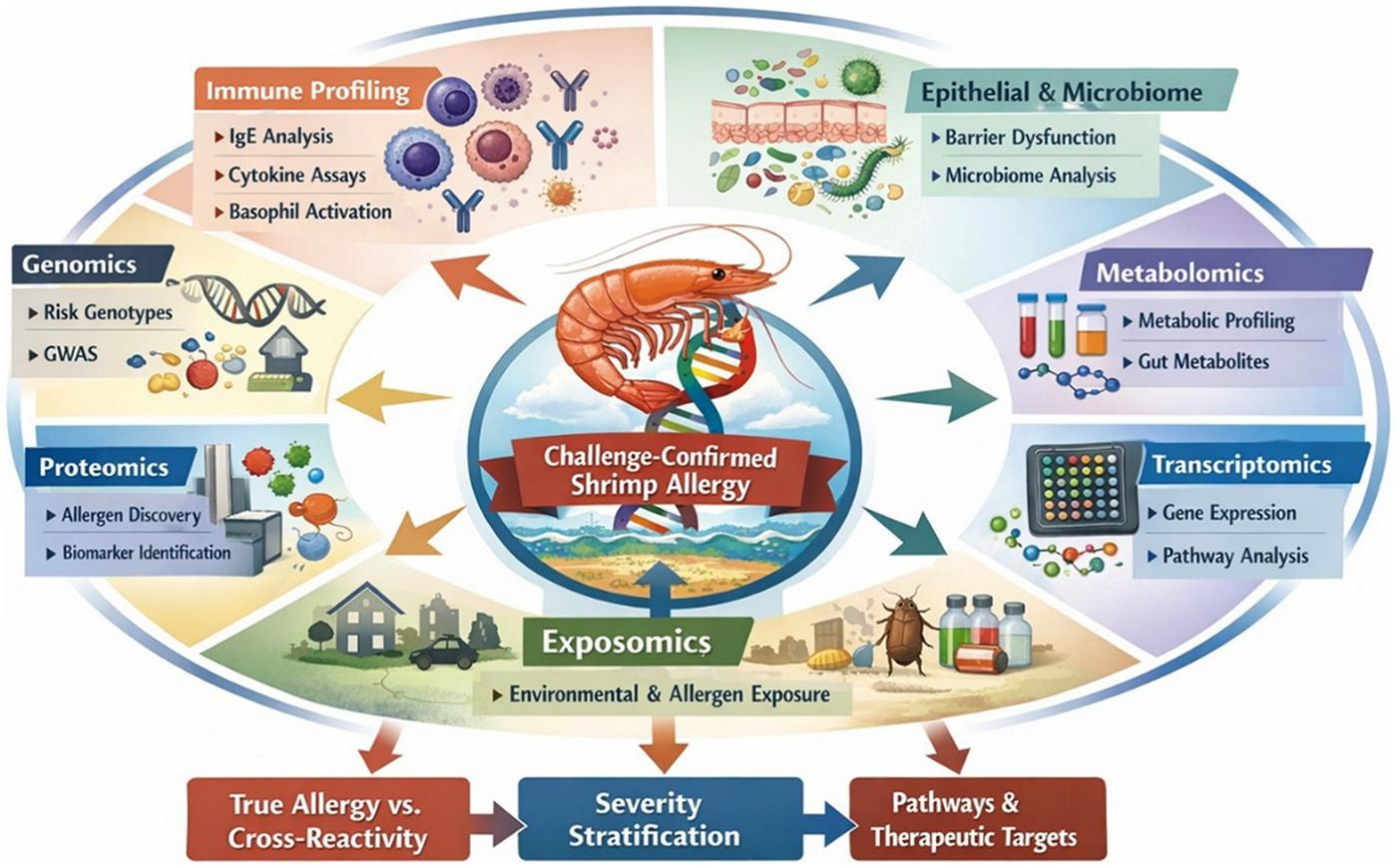
Shrimp allergy in African Americans: multi-omics and exposomics integration.

In the US, population-based surveys consistently demonstrate a higher prevalence of self-reported shellfish allergy, including SA, among African American (AA) individuals compared with non-Hispanic White populations (7). Importantly, these disparities extend beyond prevalence alone and include increased diagnostic uncertainty, higher rates of polysensitization, and disproportionate exposure to environmental risk factors that complicate accurate clinical classification (8, 9). Urban AA communities experience higher exposure to indoor allergens such as cockroach and house dust mite, as well as ambient air pollutants, which are known to influence allergic sensitization pathways (9, 10).

A central biological challenge in the diagnosis of SA in these settings is immunologic cross-reactivity [e.g., two proteins (Der p 14 and myosin heavy chain type 1) from *Dermatophagoides pteronyssinus* and *Penaeus monodon* (shrimp allergen) were identified as being common allergens]. Tropomyosin (Pen a 1), the major shrimp allergen, is highly conserved across invertebrate species and shares substantial sequence homology with tropomyosins from house dust mites and cockroaches (5, 11). As a result, shrimp-specific IgE positivity may reflect environmental sensitization rather than true clinical SA, especially in individuals with chronic pest exposure. This phenomenon can inflate estimates of shrimp sensitization and undermine the specificity of extract-based IgE testing and skin prick testing in AAs (5, 12).

Environmental exposures play a critical role in shaping allergic disease risk in urban populations, especially among AA children living in inner-city environments. Multiple studies have demonstrated disproportionately high rates of asthma, allergic sensitization, and hospitalization in these communities, driven in part by elevated exposure to indoor allergens such as cockroach and house dust mite, as well as ambient air pollutants associated with densely populated urban settings (8–10). Inner-city asthma has therefore emerged as one of the most significant allergic diseases affecting AA children and adolescents and reflects a broader pattern of environmental and structural disparities influencing allergic disease burden. Additionally, several of the environmental allergens implicated in inner-city asthma are also relevant to shrimp allergies because cockroach and dust mite tropomyosins share strong structural homology with shrimp tropomyosin, resulting in IgE cross-reactivity and potential sensitization to shrimp in individuals without direct dietary exposure (13–15). Therefore, environmental allergen exposure in urban settings may influence both the development and interpretation of shrimp sensitization, highlighting the need to consider allergic diseases including asthma, food allergy, and other atopic conditions within a shared socioenvironmental framework when evaluating biomarker performance and disease risk in AA populations.

Occupational exposure to shrimp allergens is a well-recognized cause of allergic respiratory disease among workers in seafood-processing and food-handling industries (16). During activities such as peeling, cooking, or mechanical processing, aerosolized shrimp proteins can become airborne and inhaled, leading to IgE-mediated sensitization and the development of occupational asthma or rhinitis. Studies in seafood-processing workers have demonstrated elevated rates of shrimp-specific IgE and work-related respiratory symptoms, with tropomyosin and arginine kinase identified as major inhaled allergens responsible for airway inflammation (5, 16–18). Importantly, inhalational exposure may induce sensitization even in individuals without prior dietary shrimp allergy and can subsequently predispose sensitized workers to systemic reactions following shrimp ingestion (17, 18).

In this review, we evaluate the current evidence on molecular and environmental biomarkers of SA with a specific focus on AA and African diaspora populations. We assess the strengths and limitations of existing diagnostic tools, highlight key gaps in ethnic-specific and exposure-informed research, and discuss how integrative approaches combining component-resolved diagnostics, functional assays, environmental biomarkers, and multi-omics strategies may improve diagnostic precision, and reduce misclassification in SA research.

## 2 Epidemiology of shrimp allergy

In the US, national population-based surveys estimate that approximately 1.3%–1.9% of individuals report physician-diagnosed SA, while overall shellfish allergy prevalence reaches ∼2–2.9% of adults (1, 3, 19). These estimates position shrimp as the leading crustacean allergen and a major contributor to FA burden. A recent systematic review and meta-analysis synthesizing data from more than 40 epidemiologic studies across multiple continents reported a pooled prevalence of SA of ∼1.90% based on self-reported symptoms and ∼1.94% for physician-diagnosed disease (13, 20). Importantly, prevalence estimates varied substantially by diagnostic method: studies relying on skin prick testing or serum-specific IgE reported higher pooled prevalence (∼2.7%), whereas studies incorporating clinical history with OFC (Oral Food Challenges) confirmation reported much lower estimates (∼0.4–0.5%) (13). These findings underscore the methodological heterogeneity that complicates cross-study comparisons and likely inflates prevalence estimates when challenge confirmation is absent.

Meta-analytic estimates indicate adult prevalence rates of ∼3.3–3.4% compared with ∼1.2–1.3% in pediatric populations (13, 21). U.S. survey data similarly identify shellfish allergy among the top five most common convincing FAs in adults, with shrimp accounting for nearly 2% of adult FA cases (19). Geographically, higher prevalence has been reported in parts of Asia compared with Europe and North America, likely reflecting dietary patterns, environmental exposures, and differences in diagnostic practices (5, 13). Longitudinal cohort studies show minimal rates of natural tolerance development, and epidemiologic surveys suggest that up to 50%–60% of individuals experience their first shrimp-allergic reaction in adolescence or adulthood, often after years of prior tolerance (1, 3). This late-onset phenotype is clinically consequential, as SA is strongly associated with severe outcomes: 30%–50% of affected individuals report systemic reactions or anaphylaxis, and shellfish is consistently among the leading triggers of adult food-related emergency department visits (3, 5, 22).

Analyses of US national surveys and large health-system datasets demonstrate that non-Hispanic Black individuals have significantly higher rates of shellfish and SA compared with non-Hispanic White individuals, even after adjustment for income and education (23, 24). In one large cohort study involving nearly three million patients, SA prevalence was approximately threefold higher in Black individuals than in White individuals (25). Pediatric data from the FORWARD multisite cohort further show that AA children are more likely to be allergic to shellfish and finfish and experience greater health-care utilization, including emergency department visits and hospitalizations for FA–related reactions (24, 26). Urban cohort studies provide additional insight into potential drivers of these disparities. The SAPPHIRE cohort from metropolitan Detroit reported higher rates of seafood allergy and food-associated anaphylaxis among AA participants compared with other racial and ethnic groups (25). More recently, a shrimp-focused cohort study from Detroit demonstrated that Black race, lower income, urban residence, and poor housing quality were independently associated with shrimp sensitization and clinical SA, alongside high rates of co-sensitization to cockroach and house dust mite allergens (13, 14).

These disparities are increasingly understood within a socioenvironmental framework rather than attributed to ethnicity alone. Structural inequities including historical redlining, substandard housing, pest infestation, and disproportionate exposure to air pollution are more prevalent in AA communities and are associated with elevated indoor allergen burdens (8, 26, 27). Mechanistically, this exposure context is relevant because cockroach (Bla g 7) and dust mite (*Dermatophagoides pteronyssinus*) (Der p 10, Der f 10) tropomyosins are structurally homologous to shrimp tropomyosin and exhibit extensive IgE cross-reactivity (5). Epidemiologic studies show that sensitization to inhalant tropomyosins is strongly associated with shrimp sensitization and, in a subset of Black participants from the SAPPHIRE study with available whole-genome sequencing and serum IgE data, there may be a role for inhalant-driven cross-sensitization in high-exposure urban environments (13, 14).

Despite these converging findings, major knowledge gaps persist. Most epidemiologic data in AA populations derive from cross-sectional surveys, self-reported outcomes, or extract-based diagnostics, with very few population-based studies incorporating standardized OFCs (Supplementary Table 1). As a result, current prevalence estimates likely conflate true clinical allergy with asymptomatic sensitization and cross-reactive IgE responses. Addressing these limitations will require prospective, inclusive studies that integrate standardized clinical outcomes, molecular diagnostics, and detailed environmental exposure assessment to accurately define SA burden and inform equitable precision-medicine strategies.

## 3 Molecular biomarkers

### 3.1 Allergen component–based biomarkers

Tropomyosin (Pen a 1/Pen m 1) remains the dominant and most extensively characterized shrimp (*Penaeus monodon*) allergen and represents the cornerstone of component-resolved diagnostics (CRD). Tropomyosin is a highly conserved α-helical coiled-coil muscle protein with repetitive hydrophobic amino acid pattern. This structural feature confers strong thermal and proteolytic stability, allowing the protein to persist after cooking and food processing (5, 20). Across most cohorts, 60%–80% of individuals with clinically confirmed SA exhibit IgE reactivity to tropomyosin. Moreover, higher Pen a 1–specific IgE levels are associated with positive OFCs and systemic reaction when accompanied by low allergen-specific IgG4 (4, 28).

Recognition of non-tropomyosin shrimp allergens has expanded the molecular diagnostic landscape and improved phenotypic resolution. Arginine kinase (Pen m 2), myosin light chain (Pen m 3), sarcoplasmic calcium-binding protein (Pen m 4), hemocyanin, and multiple metabolic enzymes have been identified through proteomic and transcriptomic analyses across shrimp species (2, 5, 20). These allergens differ in heat stability and immunogenicity, contributing to clinical heterogeneity. For example, arginine kinase is relatively heat labile, which may explain selective reactions to raw or lightly cooked shrimp, whereas sarcoplasmic calcium-binding protein is heat stable and has demonstrated strong basophil-activating capacity shown by in recent studied pediatric cohorts (20, 29).

Importantly, sensitization profiles incorporating multiple shrimp components, rather than tropomyosin alone appear more strongly associated with persistent and clinically relevant SA. Multi-component CRD panels that include Pen a 1 alongside non-tropomyosin allergens consistently outperform extract-based IgE testing in distinguishing true allergy from asymptomatic sensitization (3, 30). Despite the fact that cross-reactive inhalant sensitization is common in AA populations, these approaches remain underexplored in AA population, where cross-reactive inhalant sensitization is common.

### 3.2 Functional and immune mediator biomarkers

Beyond whole-molecule IgE, epitope-resolved diagnostics offer mechanistic insight into allergen recognition patterns. High-density microarrays enable mapping of IgE binding to linear and conformational epitopes across multiple shrimp allergens within a single assay (31). Distinct epitope-binding signatures have been associated with clinical reactivity and severity in other FAs, suggesting similar utility in SA. However, epitope mapping remains technically complex, resource intensive, and insufficiently validated across diverse populations (32). Recent computational analyses have further advanced epitope-level understanding, as demonstrated by Saetang et al., who used in silico approaches to identify conserved cross-reactive IgE-binding epitopes of shrimp tropomyosin shared with other arthropods, highlighting structural determinants of clinical cross-reactivity (33). Complementing this, Chen et al. performed detailed IgE epitope mapping of mantis shrimp arginine kinase and developed a hypo-immunoreactive derivative, underscoring the translational potential of epitope characterization for improved diagnostic specificity and therapeutic design (34). Ayuso et al. showed patients with positive shrimp challenges present in general more intense and diverse epitope recognition to all four shrimp allergens (35, 36).

Functional cellular assays, especially the basophil activation test (BAT), represent a promising adjunct to IgE-based diagnostics by directly quantifying allergen-induced effector cell activation through markers such as CD63 and CD203c. BAT has demonstrated superior specificity over extract-based IgE and skin prick testing for predicting shrimp OFC outcomes, largely by reducing misclassification driven by cross-reactive sensitization (37, 38). However, evidence supporting BAT in SA remains limited, as most validation studies have been conducted in Asian and Europeans with minimal representation of AA cohorts. In addition, BAT interpretation is constrained in individuals with low basophil responsiveness despite clinically confirmed allergy (39). The clinical utility and standardization of BAT in AA populations characterized by complex polysensitization profiles remain largely unexplored. Emerging complementary assays, including the mast cell activation test (MAT) and bead-based epitope assays (BBEA), are additional promise by capturing IgE-mediated activation and high-resolution epitope-specific IgE binding, respectively; however, their application to SA is similarly under-studied and requires population-specific validation (40).

Epithelial-derived IL-33 represents only one component of the broader immune cascade that drives allergic sensitization. Allergen exposure following epithelial barrier disruption can trigger the release of additional alarmins such as thymic stromal lymphopoietin (TSLP) and IL-25, which activate dendritic cells and promote type 2 immune polarization (41, 42). These antigen-presenting cells stimulate differentiation of naïve CD4+ T cells into Th2 cells that produce IL-4, IL-5, and IL-13, promoting B-cell class switching to allergen-specific IgE and recruitment of effector cells including mast cells, basophils, and eosinophils (42). Group 2 innate lymphoid cells (ILC2s), activated by epithelial alarmins including IL-33, further amplify type 2 inflammation. Thus, allergic sensitization reflects a coordinated interaction between epithelial barrier dysfunction, antigen presentation, Th2 polarization, and IgE-mediated effector responses rather than the action of IL-33 alone. Type 2 cytokines and epithelial-derived alarmins represent a mechanistically plausible but insufficiently validated class of biomarkers in SA. Although IL-33 and related Th2-associated pathways are consistently implicated across allergic diseases, their diagnostic or prognostic value in SA has not been directly established and is largely inferred from inhalant allergy and asthma studies (41, 42). These pathways may be particularly relevant in urban settings, where persistent epithelial stress and arthropod allergen exposure could modulate immune responses and contribute to inhalant–food cross-sensitization.

## 4 Environmental biomarkers

Environmental biomarkers are quantifiable indicators of exposure to external agents, such as air pollutants, indoor allergens, heavy metals, and microbiome perturbations that influence immune regulation and allergic disease risk (43). In SA, these biomarkers could reflect cumulative environmental exposures that disrupt epithelial barrier integrity, skew immune polarization toward type 2 responses, and potentially precipitate the transition from long-standing oral tolerance to IgE-mediated FA. This framework is particularly relevant given the characteristic late onset of SA and its disproportionate burden in AA populations residing in urban environments that contain elevated environmental exposures (24, 26).

Currently, direct causal evidence linking specific environmental exposures to SA remains limited, and much of the understanding is extrapolated from asthma and other atopic diseases. Nevertheless, converging mechanistic and epidemiologic data support the role of environmental factors as risk-modifying exposures that interact with molecular sensitization pathways and influence disease expression and diagnostic specificity.

### 4.1 Air pollution, indoor allergens, pest exposure, and cross-reactive sensitization

Ambient air pollutants including fine particulate matter (PM2.5), ozone (O_3_), nitrogen oxides, and polycyclic aromatic hydrocarbons (PAHs) are consistently associated with allergic disease susceptibility and severity. These pollutants induce oxidative stress, disrupt epithelial tight junctions, and activate innate immune signaling pathways that amplify type 2 inflammation (41, 44). Experimental studies demonstrate that PM2.5 and O_3_ increase epithelial permeability, facilitating allergen penetration and enhancing IgE sensitization.

PM2.5 can also function as both an allergen carrier and an immunologic adjuvant. Co-exposure to PM2.5 and ozone synergistically amplifies epithelial IL-33 release and downstream Th2 and group 2 innate lymphoid cell (ILC2) responses—pathways central to IgE class switching and allergic sensitization (42, 45, 46). Although these mechanisms are best characterized in asthma and allergic rhinitis, the same epithelial–alarmin–Th2 axis is biologically plausible in SA, particularly in individuals with compromised mucosal barriers (3, 42). Internal dose biomarkers such as urinary PAH metabolites provide quantitative measures of pollutant exposure and have been associated with increased total IgE, oxidative stress, and epithelial dysfunction—conditions that favor allergic sensitization and may predispose to more severe SA phenotypes (3, 47). In the absence of OFC–anchored longitudinal data, air pollution should be considered a risk-modifying exposure rather than a shrimp-specific causal biomarker.

Indoor allergen exposure represents the most shrimp-relevant environmental biomarker, particularly in urban AA populations. Cockroach and house dust mite allergens are highly prevalent in substandard housing and consistently elevated in predominantly AA neighborhoods (8, 48). This exposure context is critical because cockroach and dust mite tropomyosins share strong structural homology with shrimp tropomyosin, leading to IgE cross-reactivity that is associated with shrimp sensitization in some individuals, OFC–confirmed SA (13–15). Inhalant allergen–specific IgE therefore functions as a disparities-informed environmental biomarker, reflecting inhalant-driven sensitization pathways shaped by structural inequities in housing and environmental exposure (26, 27).

In high-exposure cases, this environmental context substantially reduces the specificity of shrimp extract–specific IgE testing and likely contributes to overestimation of true SA prevalence. Accordingly, indoor allergen exposure serves not only as a risk factor for sensitization but also as a critical modifier of molecular biomarker performance, reinforcing the need for component-resolved diagnostics and functional assays in these populations.

### 4.2 Heavy metals and immune dysregulation

Heavy metals, including lead, cadmium, and mercury, remain prevalent in urban environments due to aging infrastructure and industrial emissions. Even at low exposure levels, these metals exert immunotoxic effects by promoting Th2-skewed immune responses, impairing regulatory T-cell function, and disrupting antigen-presenting cell signaling (41). Epidemiologic studies consistently associate blood or urinary metal burdens with elevated total IgE levels and increased prevalence of allergic diseases, including FA (49).

In AA communities, heavy metal exposure frequently co-occurs with air pollution and indoor allergen burden, amplifying cumulative immune dysregulation shaped by structural inequities (27). Although shrimp-specific human data are sparse, the established capacity of heavy metals to disrupt immune tolerance supports their consideration as candidate environmental biomarkers within integrative SA risk frameworks. However, current evidence remains indirect and largely cross-sectional, underscoring the need for prospective studies linking metal exposure biomarkers with shrimp-specific IgE, basophil activation, and OFC outcomes.

### 4.3 Microbiome perturbation as an integrative environmental biomarker

The gut microbiome represents a key interface between environmental exposures and immune tolerance. Reduced microbial diversity and dysbiosis—shaped by urbanization, diet, antibiotic exposure, and environmental pollutants are consistently associated with FA susceptibility. Commensal taxa such as butyrate-producing *Clostridia* (clusters IV and XIVa) and *Bifidobacterium* species promote oral tolerance through regulatory T-cell induction, enhancement of epithelial barrier integrity, and production of immunomodulatory metabolites (50–52). In experimental SA models, microbiome and metabolomic perturbations modulate disease severity, while dietary interventions restore microbial homeostasis and suppress Th2 responses (53). Although human shrimp-specific data remains limited, microbiome-derived signatures represent promising integrative environmental biomarkers capturing cumulative exposure and lifestyle influences on FA risk.

Importantly these environmental biomarkers interact with host genetic susceptibility rather than acting in isolation. Variants in immune-regulatory genes such as HLA-DQ and IL-13 are associated with challenge-proven SA, and pollutant-induced epithelial injury may unmask or amplify inherited risk (54). However, most existing studies rely on cross-sectional designs and single-exposure metrics.

Overall, indoor allergen exposure, especially to cockroaches and dust mite has the strongest shrimp-specific support due to shared tropomyosin cross-reactivity and its direct impact on diagnostic specificity. Air pollution, heavy metals, and microbiome alterations currently function as biologically plausible risk modifiers rather than validated causal biomarkers. Whether mite- and cockroach-derived tropomyosins represent causal initiators of SA or simply correlate with exposures that amplify pre-existing sensitization remains a key unanswered question that, if resolved, has major implications for diagnosis and prevention strategies for SA.

## 5 Integrative approaches

The rising prevalence of FAs, coupled with the lack of robust biomarkers and disease-modifying therapies, underscores the need for integrative approaches that move beyond single-analyte diagnostics toward system-level characterization of allergic disease. In SA, however, most integrative frameworks remain largely conceptual and have not resolved the central clinical challenge of distinguishing challenge-confirmed FA from cross-reactive IgE sensitization in AAs with high environmental exposure burdens (3, 5, 14). Although emerging studies demonstrate that multi-omics integration can discriminate true allergy from cross-reactive sensitization, identify pathways underlying late-onset disease, and stratify severity risk, these approaches are most informative only when anchored to OFC–confirmed phenotypes and integrated across immune, epithelial, microbial, and environmental layers (30, 38, 55, 56). Without rigorous phenotype anchoring, omics-based integration risks reproducing the diagnostic ambiguity of extract-based IgE testing at higher dimensionality, yielding biologically rich but clinically indeterminate signatures, an issue that is consequential in AAs with prevalent background arthropod sensitization.

Proteomic and allergenomic analyses have been central to defining the shrimp allergen repertoire and understanding molecular drivers of cross-reactivity. However, while these approaches have greatly expanded the catalog of shrimp allergens, they have not yet translated into clinically validated diagnostic panels capable of reliably discriminating cross-reactive sensitization from true SA in high-exposure populations. High-resolution mass spectrometry has identified more than 20 shrimp allergen families, with tropomyosin (Pen a 1) remaining dominant but insufficient alone to define clinical allergy (5, 30). Proteomics has also enabled better understanding of cross-reactivities among allergens, suggesting that these proteins likely contribute to the cross-allergenicity observed between crustaceans and dust mites (57). Ninety proteins were differentially expressed when iTRAQ proteomics technology was used to analyse serum of shrimp and crab allergic children (58). The study further reveals differential expression of immune-related proteins associated with effector activation and inflammatory amplification, suggesting proteomic signatures that extend beyond allergen-specific IgE alone (58). Proteomic studies also highlight biologic variability in allergenicity driven by food source and processing. Natural shrimp tropomyosin demonstrates greater allergenic potency than recombinant forms, and viral infection of shrimp (e.g., white spot syndrome virus) upregulates allergenic proteins, underscoring how upstream biological context may influence downstream human immune responses (59).

Transcriptomic analyses have expanded the known shrimp allergen landscape and revealed previously unrecognized targets relevant for component-resolved diagnostics. *De novo* transcriptomic profiling across five commonly consumed shrimp species identified more than 39 previously unreported allergen candidates, many with conserved domains implicated in IgE binding and immune activation (2). In many studies, transcriptomic discovery provides a rational framework for refining diagnostic panels beyond tropomyosin, especially in populations with high background inhalant sensitization where single-component testing lacks specificity (3, 30). However, translation of transcriptomic findings into clinical diagnostics remains limited by insufficient validation in ethnically diverse, challenge-confirmed human cohorts.

Metabolomic profiling provides a functional readout of immune–microbiome interactions that shape oral tolerance. In experimental SA models, gut metabolomic shifts involving histidine, glutamate, and urocanate pathways correlate with Th2 polarization and disease severity (53). Shrimp allergic mouse stool was evaluated for gut metabolome by liquid chromatography-mass spectrometry (LC-MS). Feng et al. demonstrated that dietary or phytochemical interventions can restore microbial diversity, increase short-chain fatty acid–producing taxa, and suppress shrimp-specific Th2 responses. Human validation of these signatures remains an important gap (53).

Researchers have looked at the genes of food allergic people (especially peanut allergies). Some genes linked to FA, like FLG and STAT6, are the same as atopic dermatitis (eczema) and eosinophilic esophagitis, showing that allergic diseases may share similar immune pathways (60, 61). In West Bengal, India shrimp challenge positive people were found to have risk genotypes (i.e., HLA-DQ rs9275596 CC, IL13 rs20541 AA, and IL13 rs1800925 TT; *p* = 0.04, 0.01, and 0.03, respectively) associated with disease (54). A recent study revealed DPEP1 multiple genetic and environmental factors likely modulate allergen induced anaphylaxis, and DPEP1 as one modulator of intestinal allergen absorption (62). This and other genes may also be relevant for SA populations.

Exposomic approaches provide quantitative assessment of cumulative environmental risk, including indoor allergen burden, air pollution, metals, diet, and psychosocial stressors. In SA, indoor arthropod exposure represents the most shrimp-relevant exposomic signal due to direct molecular homology with shrimp allergens and its impact on diagnostic specificity (8, 13, 14). Studies have linked shellfish allergy with dust exposure, but the timing, dose, and causation of exposure have not been explored. Incorporating measures of cockroach and dust mite exposure alongside component-resolved diagnostics, BBEA and functional testing offers a feasible strategy for real-world clinics serving AA communities to reduce misclassification and unnecessary dietary restriction (4, 37, 55).

Despite their promise, integrative-omics approaches face significant challenges, including batch effects, platform heterogeneity, limited sample sizes, and lack of well-characterized cohorts with OFC conformed phenotypes. OFCs remain the gold standard for the diagnosis of food allergy and are essential for accurate clinical classification. However, they are resource intensive and not uniformly accessible across all clinical settings, which may contribute to diagnostic disparities in underrepresented populations (38). Machine-learning–based integrative models, successfully applied in peanut allergy to predict OFC outcomes, illustrate a potential path forward for SA, also may serve as complementary tools for risk stratification and hypothesis generation rather than substitutes for clinical evaluation (63). When used alongside standardized clinical assessment, molecular diagnostics, and functional assays, these approaches may help improve diagnostic precision and support clinical decision-making, particularly in populations with limited access to specialized allergy care.

Overall, current evidence supports a shift from reductionist biomarker discovery toward integrative, phenotype-anchored models that combine allergen components, functional immune assays, multi-omics profiling, and quantitative exposure assessment. For SA in AA populations, above approaches are essential to disentangle true FA from cross-reactive sensitization, identify pathways driving late-onset disease, and translate molecular insights into equitable clinical care (3, 55).

## 6 Future directions

Despite major advances in molecular allergology and environmental exposure science, SA research remains limited (3, 55). A critical future priority is the development of longitudinal, life-course–oriented cohorts that conceptualize SA as a dynamic process encompassing transitions between tolerance, sensitization, and clinical reactivity across childhood, adolescence and adulthood. This approach is more important given the characteristic late onset of SA after years of prior tolerance, which provides a unique opportunity to interrogate mechanisms of immune destabilization not observable in early-onset FAs (3, 5).

Future research must also move beyond single-biomarker paradigms toward systems-level causal models that integrate immune regulation, epithelial biology, and systemic metabolic pathways. Emerging evidence from multi-omics studies across allergic diseases suggests that coordinated shifts across transcriptomic, epigenomic, proteomic, and metabolomic layers better capture disease trajectories than isolated markers alone (2, 56). Applying such integrative frameworks to SA could elucidate feedback loops linking epithelial stress responses, antigen presentation, and long-lived immune memory, while also identifying compensatory or protective pathways that maintain tolerance in heavily exposed but clinically unaffected individuals. Importantly, these mechanistic models should be anchored to clinically meaningful outcomes—such as reaction thresholds, severity, and persistence—rather than sensitization alone (37, 38).

At the population level, future studies must explicitly incorporate structural and social determinants of health as upstream modifiers of biological risk. Although environmental exposures relevant to SA have been described, far less is known about how housing quality, neighborhood segregation, ambient air expopsures, food access, and chronic psychosocial stress intersect with immune pathways involved in allergic sensitization and severity (24, 26, 27). Integrating geospatial indicators, neighborhood deprivation indices, and policy-relevant exposure metrics into biologically informed study designs will be essential to disentangle socially patterned exposure from intrinsic susceptibility and to identify intervention points beyond individual-level clinical care.

Translational research represents another critical frontier. Implementation studies are needed to evaluate how emerging diagnostic and risk-stratification frameworks perform in real-world clinical settings, especially in primary care and resource-limited clinics that disproportionately serve AA communities (55). Emphasis should be placed on feasibility, interpretability, and patient-centered decision-making rather than diagnostic accuracy alone. Parallel efforts should prioritize outcome measures that reflect patient-reported burden, including quality of life, anxiety, and avoidance behaviors, which remain underrepresented in SA research despite their clear clinical relevance (3).

## 7 Conclusion

SA is a persistent and clinically significant condition that disproportionately affects AAs yet remains underrepresented in allergy research. Its diagnosis is challenging in urban, high-exposure settings where shrimp-specific IgE frequently reflects cross-reactive sensitization to cockroach and dust mite allergens rather than true clinical allergies. This limitation undermines extract-based testing and contributes to diagnostic misclassification and unnecessary dietary restriction. Emerging molecular and functional biomarkers, including multi-component and epitope-resolved diagnostics, basophil and mast cell activation assays, and bead-based epitope profiling, offer improved specificity but require validation in diverse, challenge-confirmed cohorts. Environmental and exposomic factors further shape immune dysregulation and tolerance breakdown, emphasizing that SA arises from the convergence of biological and socioenvironmental determinants (Figure 1). Future research must adopt longitudinal, multi-omics, and exposomic frameworks anchored to OFC outcomes, integrating molecular, environmental, and social determinants of health (Figure 2). Equitable, community-engaged approaches are critical to disentangle genetic susceptibility from socially patterned exposures. Such integrative, equity-centered research will refine diagnostic precision, guide personalized interventions, and advance environmental justice in SA care, ultimately transforming our understanding of late-onset FA pathogenesis and clinical management.
