# Supplementary material for: Emerging molecular and environmental biomarkers of shrimp allergy in African Americans in the US
Source: Front Allergy. 2026 Apr 10;7:1817101. doi: 10.3389/falgy.2026.1817101 (PMC13106207; doi:10.3389/falgy.2026.1817101)
Supplement: Supplementary file 1 [file Table1.docx]

Supplementary Table 1. Summary of Molecular Biomarker Classes in Shrimp Allergy

| Study / Reference | Design & Location | Sample Size (N) | Population Characteristics | Key Findings |
| --- | --- | --- | --- | --- |
| Mahdavinia et al., 2017 (NHANES) | National survey (US) | 8,890 | US children & adults, ethnically diverse | AA had significantly higher prevalence of shellfish allergy than non-Hispanic Whites; remained significant after socioeconomic adjustments. |
| Mahdavinia et al., 2021 – FORWARD Study | Prospective multi-center pediatric cohort (US) | ~2,500 | Pediatric cohort with oversampling of AA children | AA children were more likely to have shellfish allergy, higher allergic comorbidity, and increased ED visits for food-allergy reactions. |
| Davis et al., 2021 (Inner-City Cohort) | Urban clinical cohort (US) | >600 | Predominantly AA, low-income households | Extremely high cockroach allergen exposure; strong link between cockroach IgE and shrimp sensitization patterns. |
| Xiao et al., 2023 – SAPPHIRE Cohort (Detroit) | Longitudinal cohort (US) | ~4,000 | AA, White, and Hispanic adults and children | AA participants had the highest rates of seafood allergy and more severe reactions compared with other groups. |
| Zhang et al., 2025 – Detroit Shrimp Cohort | Urban shrimp-focused cohort (US) | 1,300 | Majority AA adults & adolescents | Income, urban housing quality, race, and pest exposure predicted shrimp sensitization; high co-sensitization with cockroach (Bla g 7) and dust mite (Der p 10). |
| Salo et al., 2008 – National Allergen Survey | Environmental sampling (US) | ~7,000 homes | Diverse US households; higher representation of low-income AA homes | AA households had significantly higher cockroach and dust mite allergen levels, aligning with increased shrimp sensitization risk in later studies. |
| Josey et al., 2023 (Structural Racism & Housing Study) | Policy-driven environmental health analysis | — | Urban Black communities affected by historical redlining | Demonstrated strong links between redlining, poor housing quality, and increased indoor allergen exposure, helping explain disproportionate shrimp allergy risk in AAs. |
